# Organic Trace Mineral Source Enhances the Bioavailability, Health Status, and Gut Microbiota Community in White Shrimp (*Penaeus vannamei*)

**DOI:** 10.3390/biology14050540

**Published:** 2025-05-13

**Authors:** Weijian Huang, Jinzhu Yang, Xiao Li, Gang Lin, Mingzhu Li, Yanjiao Zhang, Kangsen Mai

**Affiliations:** 1 The Key Laboratory of Aquaculture Nutrition and Feed (Ministry of Agriculture and Rural Affairs), The Key Laboratory of Mariculture (Ministry of Education), Ocean University of China, Qingdao 266003, China; 13457640693@139.com (W.H.); yangjinzhu@stu.ouc.edu.cn (J.Y.); 917639923@163.com (X.L.); kmai@ouc.edu.cn (K.M.); 2 Institute of Quality Standards and Testing Technology for Agricultural Products, Chinese Academy of Agricultural Sciences, Beijing 100081, China; ganglin.cau@gmail.com; 3 College of Agriculture, Ludong University, Yantai 264025, China; ldulimingzhu@163.com; 4 Laboratory for Marine Fisheries Science and Food Production Processes, Qingdao Marine Science and Technology Center, Qingdao 266237, China

**Keywords:** organic trace minerals, trace minerals accumulation, physiological health, intestinal microbiota, *Penaeus vannamei*

## Abstract

Trace mineral supplementation is critical for shrimp aquaculture sustainability, yet the bioavailability of conventional inorganic forms remains suboptimal. The study investigated the effects of inorganic (IM) and organic (OM) trace mineral premixes at varying doses on growth, physiological responses, and gut microbiota in white shrimp (*Penaeus vannamei*). Five experimental diets were formulated: a basal control (no supplementation), full-dose IM (IM100), half-dose IM (IM50), half-dose OM (OM50), and one-third dose OM (OM33). A total of 800 shrimp were randomly distributed into 20 tanks for an 8-week feeding experiment. The results showed that shrimp fed diets OM50 and IM100 had significantly higher total protein content in the plasma, lower levels of total cholesterol and triglyceride, and enhanced antioxidant capacity and immune response (greater activities of T-SOD, GPX, and PO, lower content of MDA, higher content of hemocyanin, and upregulated expression of *cat*, *gpx*, and *Hemo*). Furthermore, OM50 group showed superior mineral bioaccumulation, particularly for Zn in the hepatopancreas and whole body, and Cu in the whole body, accompanied by upregulated expression of mineral transporter-related (*ZIP14*, *ZnT6*, and *MT*). Moreover, diet OM50 resulted in a higher abundance of potential probiotic bacteria (including *Pseudomonas* and *Enterococcus*) and a lower abundance of potential pathogenic bacteria (including *Vibrio* and *Planctomicrobium*). The findings indicated that organic trace minerals premix at half the conventional dosage can effectively enhance its bioavailability, significantly improve the health status, and beneficially modify intestinal microbiota communities of shrimp, suggesting superior efficiency compared to inorganic forms.

## Simple Summary

Trace minerals are crucial for animal health. These include zinc, copper, manganese, iron and selenium, which are involved in various metabolic processes and enzyme composition. Excessive addition of inorganic salt forms of trace minerals in traditional feed may not only cause environmental pollution problems but also may reduce their bioavailability due to the combination of divalent cations with anti-nutritional factors (such as phytic acid, gossypol, and oxalic acid) in plant protein sources. Organic trace minerals have attracted attention due to their higher absorption rate and utilization rate. Studies have shown that they have advantages in improving the growth performance, antioxidant capacity, and immune response of aquatic animals. However, there is still a gap in the research on trace mineral premixes in white shrimp, especially in terms of apparent digestibility coefficient, tissue accumulation, and regulation of intestinal microbiota. This study aimed to explore the effects of different forms and levels of trace mineral premixes on the growth, physiological health, mineral accumulation, and gut microbiota of white shrimp, providing theoretical support for shrimp farming.

## 1. Introduction

Trace minerals are essential micronutrients for animals. Zinc (Zn) plays a key role in lipid, protein, and carbohydrate metabolism, as well as serves as a component of many enzymes, such as alkaline phosphatase (AKP), alcohol dehydrogenase, and carbonic anhydrase [1,2,3,4]. Copper (Cu) is an important oxygen carrier of hemocyanin in crustaceans and a component of Cu–Zn superoxidase (Cu-Zn SOD), lysyl oxidase, and cytochrome c oxidase [5,6]. Manganese (Mn) is necessary for proper lipid and carbohydrate metabolism and acts as an activator of glycosyltransferase, kinases, transferases, hydrolases, and decarboxylases [7,8]. A multitude of enzyme systems require iron (Fe) to maintain normal function [9,10]. Selenium (Se) is an important component of glutathione peroxidase (GPX), which together with vitamin E protects cells, tissues, and membranes from oxidative damage [11,12]. Aquatic animals mainly obtain trace minerals through their feed [13,14]. Traditionally, to meet the trace minerals requirements of animals, excessive amounts of trace minerals in the form of inorganic salts have been incorporated into commercial feeds, a practice that may pose risks for environmental contamination [8]. Simultaneously, due to the limited availability and high cost of fishmeal, there has been an increasing reliance on plant protein sources in aquafeed formulations [15,16]. However, it is important to note that plant protein sources frequently contain anti-nutritional factors such as phytic acid, gossypol, and oxalates, which can bind with divalent cations and consequently reduce the bioavailability of trace minerals to aquatic organisms [17].

Organic trace minerals, namely, metal minerals that form stable chelates through covalent or ionic bonds with amino acids, are less impacted by antinutritional compounds like phytic acid and are more readily absorbed and utilized by animals [18,19]. Multiple studies have demonstrated the enhanced efficacy of organic mineral forms. For instance, Zn–Met significantly improved growth performance and immune response in white shrimp while reducing mortality following *Vibrio harveyi* infection, compared to zinc sulfate [20]; organic Zn was also capable of promoting the carbohydrate utilization capacity of white shrimp [21]. Similarly, Cu–Met exhibited greater effectiveness than copper sulfate in enhancing antioxidant capacity, immune response, and tissue Cu accumulation in white shrimp [22] and Russian sturgeon (*Acipenser gueldenstaedtii*) [23]. Research has also indicated that organic forms of Mn and Se demonstrate superior physiological benefits compared to their inorganic counterparts [24,25,26,27].

Given that each trace mineral plays a vital role in the health and growth of aquatic organisms, dietary supplementation with multiple minerals is essential. However, research on trace mineral premixes in aquatic species is still limited, with existing studies primarily focusing on antioxidant capacity and immune responses [28,29,30,31,32]. Notably, there is insufficient research regarding the apparent digestibility coefficient (ADC) of trace minerals and their tissue accumulation. Furthermore, studies across various species, including Atlantic salmon [24,33], coho salmon (*Oncorhynchus kisutch*) [34], rainbow trout [35], striped bass (*Morone saxatih*) [36], and white shrimp [26], have reported inconsistent findings regarding minerals ADC.

The intestinal microbiota plays a crucial role in maintaining the intestinal health of the host [37]. Evidence suggests that either Zn, Cu, or Mn can modulate the relative abundance of the intestinal microbial community in animals [38,39,40]. Recent research by et al. has demonstrated that organic Mn can modify the communities of gut microbiota, thereby enhancing the antioxidant capacity and immune response in white shrimp [26]. However, only one study has investigated the effects of organic trace mineral premixes on the growth, antioxidant capacity, and immune response of white shrimp [32], indicating a significant research gap regarding mineral accumulation, mineral ADC, and intestinal microbiota.

*Penaeus vannamei* is one of the most highly produced shrimp in the world. The annual global production of farmed white shrimp has surpassed 6.5 million tons, with China ranking first in terms of production volume [41]. This study aims to conduct a comprehensive investigation into the growth performance, hematological parameters, trace mineral accumulation and ADC, antioxidant capacity, immune response, and gut microbiota of white shrimp fed diets supplemented with different forms and levels of trace mineral premixes (Zn, Cu, Mn, Fe, and Se), providing theoretical basis and technical support for the application of organic trace mineral premixes in shrimp farming.

## 2. Materials and Methods

### 2.1. Ethics Statement

Animal care and treatment procedures were approved by the Scientific Ethics Special Committee of the Academic Committee of Ocean University of China (Registration number: OUC-AE-2022-203).

### 2.2. Experimental Diets

Five isonitrogenous and isolipidic diets were formulated, and the standard levels of trace minerals of Zn, Cu, Mn, Fe, and Se were determined as 120 mg/kg, 30 mg/kg, 20 mg/kg, 30 mg/kg, and 0.3 mg/kg, respectively, based on the data provided in the published literature [12,25,42,43,44]. The basic diet formulations and trace mineral content are shown in Table 1. Control group had no addition of these five trace minerals (Control); IM100, had 100% standard levels from inorganic trace minerals (IM); IM50, had 50% standard level from IM; OM50, had 50% standard level from organic trace minerals (OM); OM33, had 33% standard level from OM. Yttrium trioxide (Y_2_O_3_), used as an inert marker, was added to all diets at a concentration of 0.1 g/kg. After all the ingredients were thoroughly mixed, the mixture was granulated to form feed pellets with a diameter of approximately 1 mm. The feed was subsequently dried for 8 h in a 55 °C ventilated oven and then stored at −20 °C until feeding.

### 2.3. Feeding Trial

White shrimp were purchased from Rizhao Tengyun Aquatic Seedlings Co., Ltd., Rizhao, China, and the feeding experiments were conducted at this company. Shrimp were fed commercial feed for one week to acclimate them to experimental housing conditions. The shrimp were starved for 24 h and weighed. A total of 800 subadult shrimp in the rapid growth stage (approximately 7 weeks old; initial body weight of 7.21 ± 0.04 g) were randomly distributed into 20 tanks each with a volume of 200 L (4 tanks/diet, 40 shrimp/tank). Shrimp were fed once daily at fixed times: 6:00, 11:00, 17:00, and 23:00. The daily feeding amount was 4% of the total body weight of the shrimp and was adjusted based on their previous feeding responses. Unconsumed feed, shrimp exuviae, and dead shrimp were removed daily at 7:00. Water quality was measured once a week during the feeding trial and maintained within the following ranges: temperature 23.8–26.3 °C, dissolved oxygen > 7.0 mg/L, ammonia < 0.2 mg/L, nitrite 0.01–0.05 mg/L, pH 7.6–8.0, salinity 18–20‰. In the water source, trace mineral concentrations were as follows: Zn < 0.001 mg/L, Cu < 0.001 mg/L, Mn 0.74 mg/L, and Fe 6.67 mg/L.

### 2.4. Sample Collection

Feces sample collection began in the fifth week and continued until the end of the 8-week feeding experiment. At approximately 13:00 daily, intact feces samples were collected via siphoning and immediately stored at −20 °C until analysis of the ADC.

Upon completion of the feeding experiment, shrimp were starved for 24 h. Survival rate was calculated, and the weight and body length of all shrimp were measured to determine the following metrics: weight gain rate (WGR), specific growth rate (SGR), condition factor (CF), feed efficiency (FE), and feed intake (FI). Twelve shrimp per tank were randomly selected for hemolymph collection from the pericardial cavity using 1 mL syringes. Hemolymph was stored in 1.5 mL Axygen tubes with anticoagulant and subsequently centrifuged at 4 °C at 4000 r/min for 10 min. The supernatant was transferred to 200 μL Axygen tubes and stored at −80 °C for hematological biochemical, antioxidant enzyme, and immune response analyses. Subsequently, the 12 shrimp were dissected on ice, and the hepatopancreas was collected for analysis of trace mineral concentrations and gene expression, the shell was collected for the analysis of trace mineral concentrations, the muscle was collected for proximate composition and trace mineral concentrations analysis, and the whole intestine was collected for the analysis of microbiota. Additionally, six shrimp per tank were collected for whole-body proximate composition and trace mineral analysis.

### 2.5. Proximate Compositions of Feeds

Analysis of the feeds chemical composition was performed according to AOAC standard protocols [45]: dry matter was measured by drying samples to a constant weight at 105 °C; crude protein was calculated by measuring nitrogen (N × 6.25) using the Kjeldahl method; crude lipid was determined by mineral ether extraction using Soxhlet method; ash content was determined by incineration of samples at 550 °C for 8 h in a muffle furnace.

### 2.6. Hematological Parameters and Enzyme Activities

Plasma concentrations of total cholesterol (T-CHO), triglyceride (TG), albumin (ALB), and malondialdehyde (MDA), as well as GPX activity, were analyzed using commercial assay kits (A111-2-1, A110-2-1, A028-2-1, A003-1-2, A005-1-2, Nanjing Jiancheng Bioengineering Institute, Nanjing, China). Plasma concentrations of glucose (GLU) and total protein (TP), and activities of acid phosphatase (ACP), AKP, and total superoxide dismutase (T-SOD), were measured with commercial kits (S0201S, P0006, P0326, P0321M, S0101M; Beyotime Biotechnology, Shanghai, China). Plasma phenoloxidase (PO) and lysozyme (LZM) activity were determined using ELISA kits (F5509-A, F952135-A; Shanghai FANKEW Industrial Co., Ltd., Shanghai, China). All parameters were analyzed according to the manufacturers’ protocols. The concentrations of hemocyanin in plasma was determined by ultraviolet absorption method, with plasma diluted into 1% solution and measured at 334 nm wavelength by ultraviolet spectrophotometer. The concentrations of hemocyanin were calculated as 2.69 times of the optical density value at 1 cm optical diameter.

### 2.7. RNA Extraction and qPCR

The total RNA of hepatopancreas was extracted using MolPure^®^ Cell/Tissue Total RNA Kit (19221ES50; Yeasen Biotechnology (Shanghai) Co., Ltd., Shanghai, China). The concentrations and quality of RNA were assessed with NanoDrop™ 2000 Spectrophotometers (Thermo Scientific™, Waltham, MA, USA). The integrity of extracted RNA was determined by electrophoresis on a 1.2% (*w*/*v*) agarose gel. The reverse transcription of 1000 ng total RNA was conducted using Evo M-MLV RT Mix Kit with gDNA Clean for qPCR Ver. 2 (AG11728; Accurate Biotechnology (Hunan) Co., Ltd., Changsha, China). The qPCR was performed in a total 20 μL volume: 3.2 μL cDNA template (≤50 ng); 0.8 μL Forward primer (10 μM); 0.8 μL Reverse primer (10 μM); 5.2 μL RNase-free ddH_2_O (P071-01, Vazyme Biotech Co., Ltd., Nanjing, China); 10 μL SYBR Green (CM0139, Accurate Biotechnology (Hunan) Co., Ltd., Changsha, China). A three-step qPCR program was used: 95 °C for 2 min, and then 40 cycles of 95 °C for 10 s, 58 °C for 10 s, and 72 °C for 20 s. At last, melting curve analysis was used to ensure the specification of the PCR product. Gene-specific primers were designed in NCBI, and synthesized by Sangon Biotech (Shanghai) Co., Ltd., Shanghai, China (Table S1). The specificity and amplification efficiency of primers were assessed. All the qPCR analysis was performed in the CFX96 Touch Real-Time PCR Detection System (Bio-Rad, Hercules, CA, USA). The gene expression levels were normalized using a relative quantitative method (2^−ΔΔCq^) referencing the β-actin of shrimp.

### 2.8. Trace Minerals Analysis

Concentrations of Zn, Cu, Mn, Fe, and Yttrium (Y) in diets, whole-body, muscle, hepatopancreas, plasma, shell, and feces were quantified by inductively coupled plasma optical emission spectroscopy (ICP-OES). Whole-body, muscle, hepatopancreas, shell, and feces samples were freeze-dried at −40 °C for 72 h, then all samples were crushed and stored at room temperature until further analysis. Approximately 0.200 g of diets, whole-body, muscle, hepatopancreas, shell, and feces samples or 100 μL plasma were weighed and transferred to digestion tube. Subsequently, 10 mL HNO_3_ (AR, 65–70%) were added to the digestion tube. The digestion process was carried out in a Multiwave 5000 microwave system (Anton Paar, Graz, Austria), with the program set as follows: preheating at 180 °C for 20 min and digestion at 180 °C for 20 min. After digestion, put the digestion tube on the acid drive machine (BHW-09A45, BOTONYC, Shanghai, China) to drive the acid (temperature 170 °C) until the digestate reached ~0.5 mL. Residues were diluted to 25 mL with ultrapure water and settled overnight. The supernatant was transferred to 15 mL centrifuge tube for preservation. All samples were analyzed for Zn, Cu, Mn, Fe, and Y using ICP-OES machine (Avio 200, Perkin Elmer, MA, USA).

### 2.9. Extraction and Sequencing of Intestinal Microbiota DNA

Genomic DNA of intestinal microbiota was extracted using QIAamp PowerFecal^®^ Pro DNA Kit (51804, Qiagen, Hilden, Germany) on a super clean bench following the manual. Primer 515F/806R was used to amplify the V4 region of the 16S rRNA gene. PCR reaction and quality control were performed by Novogene Genomics Technology Co., Ltd., Beijing, China. Sequencing was conducted on an Illumina NovaSeq platform provided by Novogene Genomics Technology Co., Ltd., Beijing, China. Based on the barcode sequence and PCR amplification primer sequence, the data of each sample was dissected from the sequencing data. After eliminating the barcode and primer sequences, the reads of the samples were concatenated using the FLASH (V1.2.11) software to obtain Raw Tags. Subsequently, the Fastp software (V0.19.4) was employed to conduct quality control on the acquired Raw Tags to generate high-quality Clean Tags. Ultimately, the Clean Tags were compared with the database via the Vsearch software (V2.23) to detect and remove chimeras, thereby obtaining the final effective data, namely Effective Tags. For the obtained Effective Tags, the DADA2 module in the QIIME2 software (V2022.11) was adopted for noise reduction, and sequences with an abundance less than five were filtered out to obtain the final Amplicon Sequence Variants (ASVs) and their feature table. Next, the ASVs were compared with the database using the classify-sklearn module in the QIIME2 software to obtain the species information corresponding to each ASV. The QIIME2 software was utilized to calculate ASVs, Chao1, good_coverage, Pielou, Shannon, and Simpson indices, and a dilution curve and species accumulation boxplot were drawn. The Unifrac distance was calculated with the QIIME2 software, and a PCoA dimensionality reduction plot drawn with the aid of the R (V3.5.3) software, which invokes the *ade4* (V1.7-23) and *ggplot2* (V3.5.1) packages. At the genus classification level, MetaStat analysis was conducted on the two comparison groups, and the difference test is executed using the R software to obtain *p*-values. Genera with *p*-values less than 0.05 were screened out as significantly different species among the groups.

### 2.10. Calculations

The equations for growth performance as follows:

WGR (%) = 100 × (final body weight − initial body weight)/initial body weight;

SGR (%/day) = 100 × (Ln (final body weight) − Ln (initial body weight))/days;

CF (100 g/cm^3^) = 100 × final body weight/final body length^3^;

FI (%/day) = 100 × feed intake/[(initial body weight + final body weight)/2]/days;

FE = (final body weight − initial body weight)/feeds consumed;

Survival (%) = 100 × final number of shrimp/initial number of shrimp.

The equations for chemical compositions as follow:

Moisture (%) = (wet weight − dry weight)/wet weight × 100;

Crude ash (%) = ash weight/dry weight × 100.

The equation for ADC of minerals as follows:

ADC (%) = 100 × [1 − (mineral in feces × Y_2_O_3_ in feeds)/(mineral in feeds × Y_2_O_3_ in feces)]

### 2.11. Statistical Analysis

Results were analyzed by one-way analysis of variance (ANOVA). When ANOVA identified significant differences among groups (*p* < 0.05), multiple comparisons were performed using Tukey’s multiple range test. All statistical analyses were conducted with SPSS 27.0.1 for Windows (IBM Corp., Armonk, NY, USA). Data are expressed as means ± S.E.

The correlation analysis of trace minerals (Zn, Cu, Mn, and Fe) among various tissues and the correlation analysis of trace minerals (Zn, Cu, Mn, and Fe) in the hepatopancreas with enzymatic activities (T-SOD, GPX, MDA, ACP, AKP, PO, LZM, and hemocyanin) in plasma and gene expressions (*cat*, *gpx*, *acp*, *akp*, *proPO*, *hemo*, *ZIP1*, *ZIP11*, *ZIP14*, *ZnT2*, *ZnT6*, *ZnT7*, *Ctr1*, *ATOX1*, *ATP7b*, and *MT*) in the hepatopancreas were performed using the R package *corrplot*. In the correlation analysis of trace minerals, the samples were classified into inorganic trace mineral group (IM, consisting of the control, IM100, and IM50) and organic trace mineral group (OM, consisting of the control, OM50, and OM33) for analysis.

## 3. Results

### 3.1. Growth Performance

There were no significant differences in FBW, WGR, SGR, CF, FI, FE, survival rate, crude protein, crude lipid, and crude ash of shrimp observed among all groups during the 8-week feeding period (*p* > 0.05) (Table 2).

### 3.2. Hematological Parameters of Plasma

The T-CHO content was significantly lower in the IM100, OM50, and OM33 groups than in the control group (*p* < 0.05). Plasma TG content was highest in the control group, and the TG content in the IM50 group was significantly higher than those of the IM100, OM50, and OM33 groups (*p* < 0.05). No significant differences in GLU content were observed among groups (*p* > 0.05). The TP content was significantly higher in the IM100 and OM50 groups compared to the control, IM50, and OM33 groups; the TP content in the IM50 group was significantly higher than that in the control group (*p* < 0.05). ALB content in the IM100 and OM50 groups was significantly higher than in the OM33 group (*p* < 0.05) (Table 3).

### 3.3. Mineral (Zn, Cu, Mn, and Fe) Accumulation in Various Tissues

**Figure 1:**
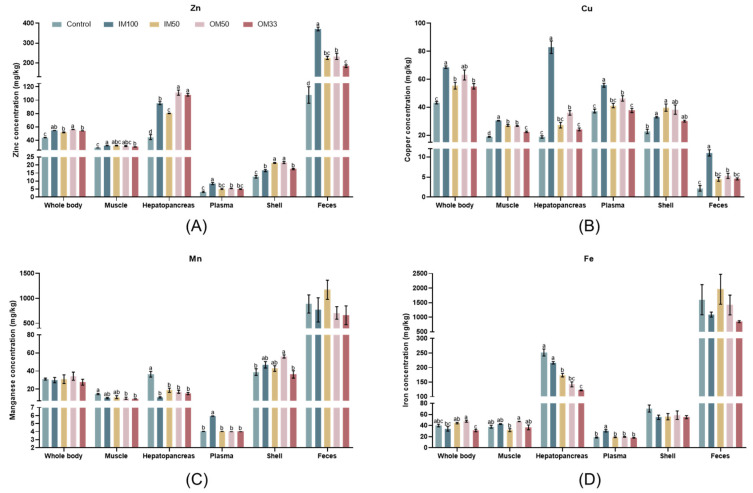
Zn (**A**), Cu (**B**), Mn (**C**), and Fe (**D**) accumulation in the whole body, muscle, hepatopancreas, plasma, shell, and feces of white shrimp fed with organic or inorganic trace minerals premixes. Results are shown as means ± S.E. of four replicates. Different superscript letters indicate significant differences (*p* < 0.05).

As shown in Figure 1A, the shrimp treated with OM50 exhibited the highest whole-body Zn concentrations when compared to the control, IM50, and OM33 groups. The IM50 and OM33 groups demonstrated higher whole-body Zn concentrations than the control group (*p* < 0.05). The muscle Zn concentrations in the IM100 group was significantly higher compared to the control and OM33 groups (*p* < 0.05). The OM50 and OM33 groups had significantly higher hepatopancreas Zn concentrations compared to the control, IM100, and IM50 groups; the hepatopancreas Zn concentrations of the IM100 group was significantly higher than the control and IM50 groups (*p* < 0.05). The IM100 group had the highest Zn concentrations in the plasma and feces (*p* < 0.05). The shell Zn concentrations in the shrimp receiving IM50 and OM50 diets was significantly higher compared to the control, IM100, and OM33 groups; the shell Zn concentrations of the IM100 and OM33 groups was significantly higher than the control group (*p* < 0.05).

The shrimp treated with IM100 diet had higher whole-body Cu concentrations compared to the control, IM50, and OM33 groups. The OM50 group demonstrated higher whole-body Cu concentrations than the control group (*p* < 0.05). The shrimp treated with IM100 had the highest Cu concentrations in the muscle, hepatopancreas, plasma, and feces. The OM50 group had significantly higher Cu concentrations in the muscle, hepatopancreas, and plasma compared to the control and OM33 groups (*p* < 0.05). The shell Cu concentrations in the IM100 and IM50 groups was higher compared to the control group (*p* < 0.05) (Figure 1B).

There was no significant difference in the Mn concentrations of whole body or feces among all groups (*p* > 0.05). The Mn concentrations of muscle was significantly higher in the control group compared to the OM50 and OM33 groups (*p* < 0.05). The control group had the highest hepatopancreas Mn concentrations (*p* < 0.05). The IM100 group had the highest plasma Mn concentrations (*p* < 0.05). The shell Mn concentrations was significantly higher in the OM50 group compared to the control and OM33 groups (*p* < 0.05) (Figure 1C).

The whole-body Fe concentrations was significantly higher in the OM50 group compared to the IM100 and OM33 groups (*p* < 0.05). The muscle Fe concentrations was significantly higher in the OM50 group compared to the IM50 group (*p* < 0.05). The hepatopancreas Fe concentrations was significantly higher in control and IM100 groups compared to the IM50, OM50, and OM33 groups (*p* < 0.05). The IM100 group had the highest plasma Fe concentrations (*p* < 0.05). There was no significant difference in the shell and feces Fe concentrations among all groups (*p* > 0.05) (Figure 1D). The values of minerals (Zn, Cu, Mn, and Fe) concentrations are shown in Table S2.

### 3.4. Apparent Digestibility Coefficient of Trace Minerals

The ADC of Zn was significantly greater in control, IM50, and OM50 groups compared to the IM100 group (*p* < 0.05). The ADC of Cu was significantly greater in the IM50, OM50, and OM33 groups compared to the control group (*p* < 0.05). The values of ADC of Zn, Cu, Mn, and Fe are shown in Table 4.

### 3.5. The Expression of Metal Transporter Genes

**Figure 2:**
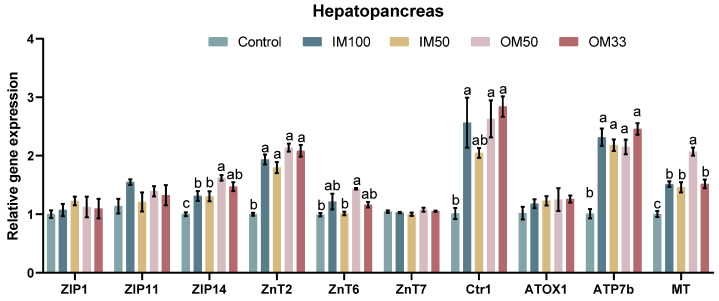
Metal transport genes in the hepatopancreas of white shrimp fed with organic or inorganic trace minerals premixes. Results are shown as means ± S.E. of four replicates. Different superscript letters indicate significant differences (*p* < 0.05).

In the hepatopancreas, the expression of *ZIP14* and *MT* was significantly upregulated in the OM50 group compared to the control, IM100, and IM50 groups (*p* < 0.05). The expression of *ZnT2* and *ATP7b* was significantly downregulated in the control group compared to other groups (*p* < 0.05). There were no significant differences in the *ZIP1*, *ZIP11*, *ZnT7*, and *ATOX1* expression among all groups (*p* > 0.05) (Figure 2). The values of the expression levels of metal transporter genes are shown in Table S3.

### 3.6. Antioxidant Capacity

**Figure 3:**
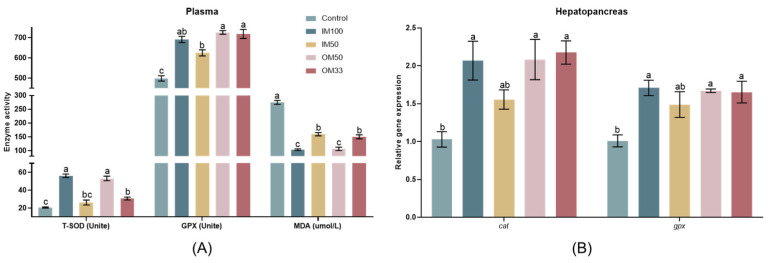
Antioxidant enzyme in plasma (**A**) and the expression of antioxidant response genes in the hepatopancreas (**B**) of white shrimp fed with organic or inorganic trace minerals premixes. Results are shown as means ± S.E. of four replicates. Different superscript letters indicate significant differences (*p* < 0.05).

In the plasma of white shrimp, the IM100, OM50, and OM33 groups had higher T-SOD activities compared to the control group (*p* < 0.05). The OM50 and OM33 groups had higher GPX activities compared to the control and IM50 groups (*p* < 0.05). The IM100 and OM50 groups had lower MDA concentrations compared to the control, IM50, and OM33 groups (*p* < 0.05) (Figure 3A).

In the hepatopancreas of white shrimp, the expressions of *cat* and *gpx* were significantly upregulated in the IM100, OM50, and OM33 groups compared to the control group (*p* < 0.05) (Figure 3B). The values of antioxidant capacity are shown in Table S4.

### 3.7. Immune Response

**Figure 4:**
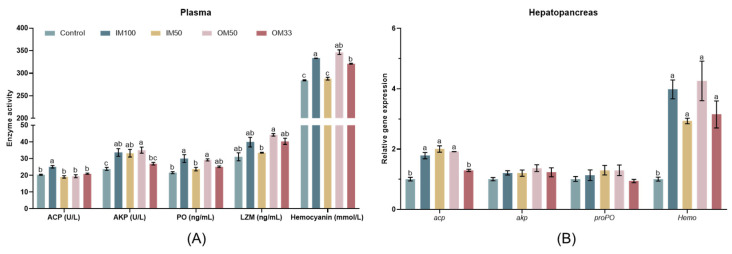
Immune response in plasma (**A**) and the expression of immune response genes in the hepatopancreas (**B**) of white shrimp fed with organic or inorganic trace minerals premixes. Results are shown as means ± S.E. of four replicates. Different superscript letters indicate significant differences (*p* < 0.05).

In the plasma of white shrimp, the IM100 group had the highest ACP activities (*p* < 0.05). The IM100, IM50, and OM50 groups had higher AKP activities compared to the control group (*p* < 0.05). The IM100 and OM50 groups had higher PO activities compared to the control and IM50 groups (*p* < 0.05). The OM50 group had higher LZM activities compared to the IM50 group (*p* < 0.05). The IM100 and OM50 groups had higher hemocyanin content compared to the control and IM50 groups (*p* < 0.05) (Figure 4A).

In the hepatopancreas of white shrimp, the expression of *acp* significantly upregulated in the IM100, IM50, and OM50 groups compared to the control and OM33 groups (*p* < 0.05). There was no significant difference in the expression levels of *akp* and *proPO* among all groups (*p* > 0.05). The expression of *Hemo* significantly upregulated in the IM100, IM50, OM50, and OM33 groups compared to the control group (*p* < 0.05) (Figure 4B). The values of immune response indicators are shown in Table S5.

### 3.8. Correlation Analysis

**Figure 5:**
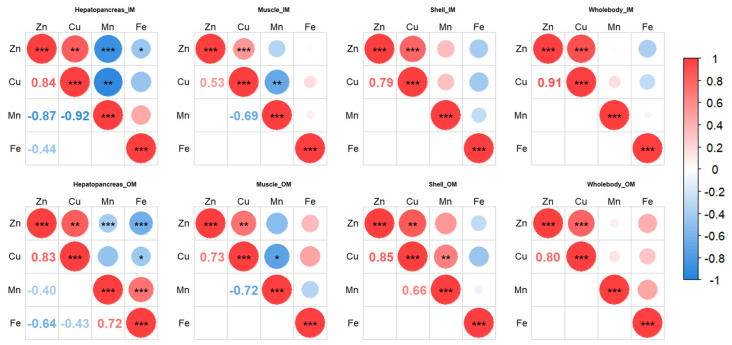
Spearman’s correlation analysis of Zn, Cu, Mn, and Fe in the hepatopancreas, muscle, shell, and whole body of white shrimp. Spearman’s correlation coefficient (*r*) is shown in the cells. The size of the circle indicates the strength of the correlation. *, **, ***: indicates that the *p*-value is less than 0.05, 0.01, 0.001, respectively. IM: consisting of the control, IM100, and IM50. OM: consisting of the control, OM50, and OM33.

In the hepatopancreas, there were positive correlations between I-Zn and I-Cu (Inorganic trace minerals group, *r* = 0.84, *p* < 0.01), O-Zn and O-Cu (Organic trace minerals group, *r* = 0.83, *p* < 0.01), and O-Mn and O-Fe (*r* = 0.72, *p* < 0.001). There were negative correlations between I-Zn and I-Mn (*r* = −0.87, *p* < 0.001), I-Zn and I-Fe (*r* = −0.44, *p* < 0.05), I-Cu and I-Mn (*r* = −0.92, *p* < 0.01), O-Zn and O-Mn (*r* = −0.43, *p* < 0.001), O-Zn and O-Fe (*r* = −0.64, *p* < 0.001), and O-Cu and O-Fe (*r* = −0.40, *p* < 0.05). In the muscle, there were positive correlations between I-Zn and I-Cu (*r* = 0.53, *p* < 0.001) and O-Zn and O-Cu (*r* = 0.73, *p* < 0.01). There were negative correlations between I-Cu and I-Mn (*r* = −0.69, *p* < 0.01) and O-Cu and O-Mn (*r* = −0.72, *p* < 0.05). In the shell, there were positive correlations between I-Zn and I-Cu (*r* = 0.79, *p* < 0.001), O-Zn and O-Cu (*r* = 0.85, *p* < 0.01), and O-Cu and O-Mn (*r* = 0.66, *p* < 0.01). In the whole body, there were positive correlations between I-Zn and I-Cu (*r* = 0.91, *p* < 0.001), and O-Zn and O-Cu (*r* = 0.80, *p* < 0.001) (Figure 5).

**Figure 6:**
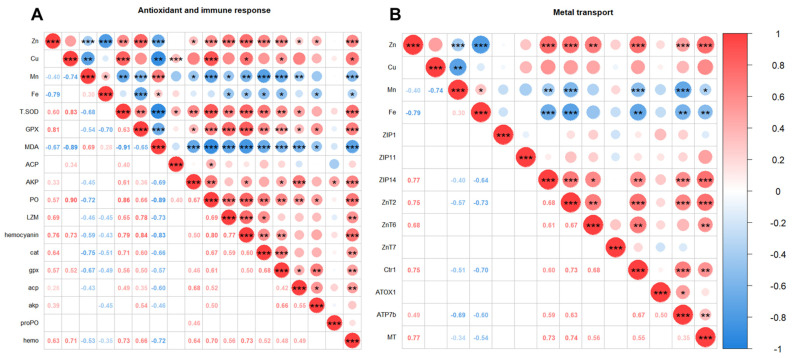
Spearman’s correlation analysis of Zn, Cu, Mn, and Fe in the hepatopancreas and the antioxidant and immune response (**A**) and the expression of metal transporter genes in hepatopancreas (**B**). Spearman’s correlation coefficient (*r*) is shown in the cells. The size of the circle indicates the strength of the correlation. *, **, ***: Indicates that the *p*-value is less than 0.05, 0.01, 0.001, respectively.

Spearman’s correlation analysis of Zn, Cu, Mn, and Fe in the hepatopancreas and the activities of antioxidant and immune enzymes in plasma, the expression of antioxidant and immune genes in hepatopancreas, and the expression of metal transporter genes in hepatopancreas are shown in Figure 6. Examining mineral-enzyme relationships (Figure 6A), there were positive correlations between Zn content and T-SOD (*r* = 0.60, *p* < 0.01), GPX (*r* = 0.81, *p* < 0.001), AKP (*r* = 0.33, *p* < 0.05), PO (*r* = 0.57, *p* < 0.001), LZM (*r* = 0.69, *p* < 0.001), and hemocyanin (*r* = 0.76, *p* < 0.001). For Cu positive correlations were observed with T-SOD (*r* = 0.83, *p* < 0.001), ACP (*r* = 0.34, *p* < 0.001), PO (*r* = 0.90, *p* < 0.001), and hemocyanin (*r* = 0.73, *p* < 0.05). There were negative correlations between Zn and MDA (*r* = −0.67, *p* < 0.001), as well as Cu and MDA (*r* = -0.89, *p* < 0.01). Negative correlations with Mn were found for T-SOD (*r* = −0.68, *p* < 0.01), GPX (*r* = −0.54, *p* < 0.001), AKP (*r* = −0.45, *p* < 0.05), PO (*r* = −0.72, *p* < 0.001), LZM (*r* = −0.46, *p* < 0.05), and hemocyanin (*r* = −0.59, *p* < 0.01). Fe had negative correlations with GPX (*r* = −0.70, *p* < 0.001), LZM (*r* = −0.45, *p* < 0.05), and hemocyanin (*r* = −0.43, *p* < 0.05).

For gene expression, there were positive correlations between Zn and *cat* (*r* = 0.64, *p* < 0.001), *gpx* (*r* = 0.57, *p* < 0.001), *acp* (*r* = 0.26, *p* < 0.05), *akp* (*r* = 0.39, *p* < 0.05), and *hemo* (*r* = 0.63, *p* < 0.001). While Cu was positively correlated with *gpx* (*r* = 0.52, *p* < 0.05) and *hemo* (*r* = 0.71, *p* < 0.05). There were negative correlations between Mn and *cat* (*r* = −0.75, *p* < 0.001), *gpx* (*r* = −0.67, *p* < 0.001), *acp* (*r* = −0.43, *p* < 0.01), and *hemo* (*r* = −0.53, *p* < 0.001), as well as Fe and *cat* (*r* = −0.51, *p* < 0.05), *gpx* (*r* = −0.49, *p* < 0.05), *akp* (*r* = −0.45, *p* < 0.05), and *hemo* (*r* = −0.35, *p* < 0.05) (Figure 6A).

Metal transporter genes showing positive correlation with Zn included *ZIP14* (*r* = 0.77, *p* < 0.001), *ZnT2* (*r* = 0.75, *p* < 0.001), *ZnT6* (*r* = 0.68, *p* < 0.01), and *MT* (*r* = 0.77, *p* < 0.001). While there were negative correlations between Mn and *ZIP14* (*r* = −0.40, *p* < 0.01), Mn and *MT* (*r* = −0.34, *p* < 0.05), Fe and *ZIP14* (*r* = −0.64, *p* < 0.001), and Fe and *MT* (*r* = −0.54, *p* < 0.01) (Figure 6B).

### 3.9. Intestinal Microbiota

**Figure 7:**
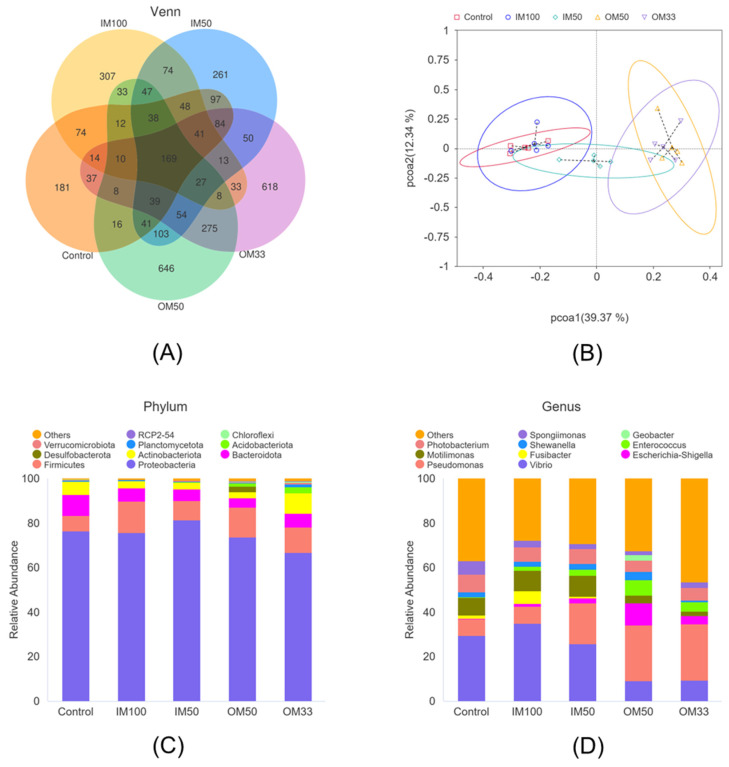
Effects of organic and inorganic trace minerals on intestinal microbiota of white shrimp. Flower diagram of intestinal microbiota among all groups (**A**). Principal coordinate analysis (PCoA) plot in samples (**B**) based on bray_curtis distances among all groups. Taxonomy classification of reads at phylum (**C**) and genus (**D**) levels. Only the top 10 most abundant (based on relative abundance) bacterial phyla and genera were shown in the figures; other phyla and genera were all assigned as ‘Others’.

After annotation, a total of 42 phyla, 105 classes, 253 orders, 436 families, 972 genera, and 1832 ASVs were identified. Both the rarefaction curves and the species accumulation boxplot approached saturation, suggesting that the sequencing depth of the sample was adequate (Figure S1). The Venn diagram reveals that all groups share a total of 169 ASVs, and the control, IM100, IM50, OM50, and OM33 group possess 181, 307, 261, 646, and 618 unique ASVs, respectively (Figure 7A). The results of the principal coordinate analysis (PCoA) disclosed that the distance between the OM50 and OM33 groups was relatively short, and these two groups were distinctly segregated from the control and IM100 groups (Figure 7B). The results of the alpha diversity analysis indicated that the ASVs and Chao1 indices of the IM50 and OM50 groups were significantly higher than those of the control group, whereas the goods_coverage was significantly lower than that of the control group (*p* < 0.05). No significant differences were manifested in the pielou_e, Shannon, and Simpson indices among all groups (*p* > 0.05) (Table S6).

**Figure 8:**
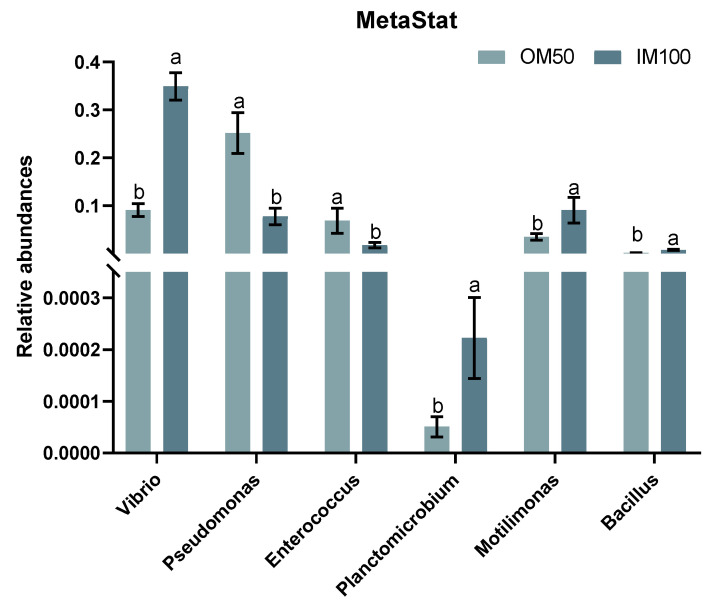
Meta-statistical analysis of the intestinal microbiota of white shrimp at the genus level between the OM50 and IM100 group. Results are shown as means ± S.E. of four replicates. Different superscript letters indicate significant differences (*p* < 0.05).

At the phylum level, Proteobacteria, Firmicutes, Bacteroidetes, and Actinobacteria are the four major bacterial phyla in the shrimp intestinal (Figure 7C). At the genus level, *Vibrio*, *Pseudomonas*, *Escherichia–Schigella*, *Fusibacter*, *Motilimonas*, *Enterococcus*, *Shewanella*, *Photobacterium*, *Geobacter*, and *Spongiimonas* were the predominant in the intestine among all groups (Figure 7D). In the MetaStat analysis at the genus level, the results demonstrated that the relative abundances of *Pseudomonas* and *Enterococcus* were significantly higher in the OM50 group compared with the IM100 group, while the relative abundances of *Vibrio* and *Planctomicrobium* were significantly lower (Figure 8). In contrast to the control group, 21, 67, 74, and 64 differentially abundant bacterial genera were respectively observed in the IM100, the IM50, the OM50, and the OM33 groups (Figure S2).

## 4. Discussion

In this study, different forms of trace mineral premixes exerted no significant effects on the growth performance of white shrimp, consistent with findings in rainbow trout [29,46], gilthead seabream [30], and rockfish [31]. However, Katya et al. reported that organic trace minerals premixes (Cu, Zn, and Mn) enhanced growth in white shrimp [32]. This discrepancy may relate to differences in shrimp life stages: their study used juveniles with an IBW of 0.6 g (high growth plasticity), whereas our trial involved larger shrimp (IBW of 7.21 g), which may exhibit reduced sensitivity to trace mineral supplementation. Similarly, organic Cu improved the growth of white shrimp with an IBW of 1.86 g [47], but did not find a growth difference of white shrimp with an IBW of 5.30 g [22]. These and other discrepancies in research results also likely arise from variations in species, environmental conditions, and the composition of their basic diet. This highlighted the importance of reporting such parameters to allow accurate comparison and build understanding of how these factors interact with nutritional needs.

The nutritional and health status of aquatic animals is frequently assessed through hematological and biochemical indicators [48]. Extra high plasma T-CHO and TG levels are usually associated with the occurrence of diseases [49]. In this study, dietary supplementation with trace minerals—particularly in the IM100 and OM50 groups—significantly reduced plasma T-CHO and TG levels. This effect may be linked to the sufficient availability of Zn and Cu. Studies on shrimp had revealed increasing Zn levels in diet, activates the Zn transporter and the Ca^2+^/CaMKK*β*/AMPK pathway, reducing lipid-related parameters (e.g., T-CHO, HDL-cholesterol, and nonestesterified fatty acid) in hepatopancreas [42], and dietary Cu had been shown to regulate lipid metabolism and promote β-oxidation, significantly enhancing the energy generation and lipolytic capacity of white shrimp [50]. These findings suggested that both Zn and Cu play crucial roles in modulating lipid metabolism and energy balance in shrimp. Furthermore, the supplementation of trace minerals, particularly the diet OM50, resulted in higher plasma TP, which is advantageous for shrimp growth and immune responses [51].

Under the present experimental conditions, dietary trace mineral sources (organic vs. inorganic) significantly influenced Zn and Cu accumulation in shrimp tissues. In shrimp, the hepatopancreas is the main organ for mineral metabolism and storage [52], with higher mineral content in the hepatopancreas indicating higher bioavailability of minerals [46]. The homeostasis of Zn^2+^ is controlled by the coordination of the SLC39 family (ZIPs), the SLC30 family (ZnTs), and MT [53]. The SLC39 family is responsible for Zn^2+^ transport from the extracellular or organelles to the cytoplasm, while the SLC30 family performs the opposite function [54]. MT has a high affinity for various metals (e.g., Zn^2+^, Cu^2+^) and contributes to ion homeostasis [55,56]. In the present study, shrimp fed with organic trace minerals exhibited significantly higher expression of hepatopancreas Zn transport genes (*ZIP14* and *ZnT6*, and *MT*), as well as greater hepatopancreas and whole-body Zn content. These findings indicate that the bioavailability of organic zinc was superior to that of inorganic Zn, which is consistent with previous studies on organic trace minerals (Cu, Zn and Mn) in white shrimp [32]. Similarly, research on marine rockfish demonstrated that organic trace minerals (Cu, Zn, Mn, and Fe) substantially enhanced hepatopancreatic Zn levels compared to their inorganic counterparts [31]. This phenomenon may be attributed to the stable structure of organic Zn, which facilitates transport in an intact form and minimizes interactions with anti-nutritional factors such as phytic acid [57]. Furthermore, a pronounced positive correlation was detected between hepatopancreas Zn content and the expression of *ZIP14*, *ZnT6*, and *MT*, suggesting their active involvement in Zn absorption and accumulation. Copper absorption is primarily mediated by Ctr1 on the cell membrane [58]; ATOX1, a Cu chaperone protein, regulates intracellular Cu transport and distribution [59], while ATP7b exhibits Cu-dependent translocation in response to elevated intracellular Cu concentrations [60]. In this study, trace minerals supplementation significantly upregulated the expression of Cu transporters (*Ctr1*, *ATP7b*, and *MT*) compared to the control group; however, no significant differences were observed between organic and inorganic forms. Perhaps it is attributable to the fact that there are similar critical steps or restrictive factors in the processes of inorganic and organic forms of Cu entering cells, undergoing metabolic transformation, or interacting with other molecules, that the regulation of Cu transporter expression by them tends to be uniform [61]. The lack of significant differences in *ATOX1* expression across groups might relate to Cu storage dynamics. Goff proposed that abundant Cu reserves promote MT synthesis in intestinal epithelial cells, causing Cu^2+^ to preferentially bind to *MT* rather than *ATOX1* [62]. The observed increase in Cu content in whole-body, muscle, hepatopancreas, and plasma supports this hypothesis and aligns with Katya et al. [32]. Elevated Cu levels enhance antioxidant capacity and immune responses in shrimp, as Cu serves as a cofactor for enzymes involved in cellular respiration and antioxidant defense [63]. Excessive mineral excretion can result in the deterioration of water quality and have negative effects on the environment [64]. Notably, the OM50 diet not only improved shrimp health but also resulted in significantly lower Zn and Cu excretion. This indicates that organic trace minerals can meet nutritional requirements while reducing environmental pollution risks associated with mineral discharge.

In this study, dietary trace minerals supplementation did not affect Mn levels in the whole-body, muscle, or hepatopancreas, nor did it reveal a discernible trend in Fe content. The competitive absorption of Zn may hinder Mn and Fe uptake due to share transport mechanisms [65]. Alternatively, minerals might compete by activating opposing enzyme systems; for example, copper activates ascorbate oxidase to oxidize ascorbic acid, whereas Mn stimulates lactose synthesis to promote vitamin production [66]. Similar to the present results, different levels of trace mineral premixes exerted no significant effect on hepatopancreas Mn content in white shrimp [32], and no significant differences were manifested in whole-body Mn and Fe contents in marine rockfish [31]. In contrast, research on cobia (*Rachycentron canadum*) have demonstrated that increased Mn supplementation led to elevated Mn content in the whole-body and vertebrae [67]. The observed discrepancies urgently require in-depth investigation, incorporating multiple factors such as species-specific physiology, trace mineral dosages/formulations, experimental conditions, and metabolic regulation pathways.

The ADC serves as a critical indicator for evaluating nutrient absorption, utilization, and nutritional regimen optimization. In this study, zinc ADC values ranged from 11.68% to 25.91%, consistent with findings in white shrimp [26]. Notably, the IM100 group exhibited the lowest Zn ADC, likely due to its elevated dietary Zn content of 155.17 mg/kg, which substantially exceeded the established requirement of 104.8 mg/kg for white shrimp [50]. This observation aligns with previous research showing that phosphorus supplementation beyond requirements reduces phosphorus ADC [68]. A similar relationship for other minerals, such as Zn and Cu, would not be unexpected. In line with this, the Cu ADC value of the IM100 group was also lower than that of the other groups with trace mineral supplementation. Interestingly, both Mn and Fe ADCs yielded negative values in this study, mirroring reports in white shrimp [26], rainbow trout [24], and Atlantic salmon [33]. This may be attributed to either the absorption of Mn and Fe by shrimp from seawater, leading to an elevated content in feces, or the fact that the contents of Mn and Fe in the feed surpassed the requirements of the shrimp. And feed composition, minerals forms, mineral-mineral interactions, and mineral-antinutrient relationships all influence mineral ADC [33]. For instance, studies on white shrimp and Atlantic salmon have shown an inverse correlation between dietary Mn and Cu ADC [24,26]. Thus, comprehensive understanding of the properties and proportions of various components in the feed is highly significant, which can ensure their rational combination and minimize unfavorable interactions. Although Se analysis was constrained by low supplementation levels, its biological relevance emerged through GPX activity. As a key GPX component, Se synergizes with vitamin E to protect cells from oxidative damage [62]. In the present study, organic trace minerals treatments displayed the highest GPX activity and *gpx* expression, indicating superior absorption and bioavailability of organic versus inorganic Se.

The interactions among trace minerals play crucial roles in maintaining normal physiological functions through synergistic and antagonistic relationships. While Zn and Cu exhibit known antagonism—excessive Zn inhibits Cu absorption and vice versa [62] 2013 our findings revealed significant positive Zn-Cu correlations across hepatopancreas, muscle, shell, and whole-body. Notably, organic trace minerals exhibited stronger Zn-Cu correlations (muscle: OM *r* = 0.85 vs IM *r* = 0.53; shell: OM *r* = 0.85 vs IM *r* = 0.79), which may be related to less competition or interaction through organic chelation [69]. In the hepatopancreas, Zn and Cu levels correlated positively with T-SOD, PO, and hemocyanin activities, as well as *gpx* and *hemo* expressions, suggesting their synergistic effects in maintaining physiological functions through regulating antioxidant and immune parameters. Results shown a negative correlation between Zn and Mn, Zn and Fe, as well as Cu and Mn in the hepatopancreas, aligning with the report of Goff [62]. This may stem from ZIP transporter substrate specificity, which mediates the transport of metal ions such as Zn, Mn, and Fe into cells. Our data demonstrated that *ZIP14* levels associated positively with Zn (*r* = 0.77) but negatively with Mn (*r* = −0.40) and Fe (*r* = −0.64), proposing competitive absorption via shared transport pathways. Studies on Nile tilapia, yellow catfish, and rainbow trout also show that the addition of Zn in feed can reduce the Fe content in tissues [70,71,72]. Understanding these mineral interrelationships has significant implications for advancing our knowledge in nutrition, physiology, and health management.

In the present study, hepatopancreatic trace minerals analysis revealed strong positive correlations between Zn/Cu content and antioxidant/immune responses, whereas Mn/Fe showed predominantly negative correlations. These findings suggest Zn and Cu play pivotal roles in maintaining shrimp health and oxidative balance. The T-SOD, GPX, and MDA are established biomarkers of crustaceans antioxidant capacity [73]. The supplementation of trace minerals significantly enhanced shrimp antioxidant capacity compared to the control group, as evidenced by elevated T-SOD and GPX activity, and reduced MDA levels. This improvement likely originated from increased levels of antioxidant minerals such as Zn, Cu, Mn, and Se, which are established cofactors for antioxidant enzymes like Cu/Zn SOD and GPX [11,74]. Notably, half-dose organic minerals (OM50) achieved antioxidant efficacy comparable to full-dose inorganic minerals (IM100), demonstrating superior bioavailability of organic forms. The antioxidant system interacts synergistically with immune responses to maintain homeostasis, forming an integrated defense network [74]. In present study, organic trace minerals enhanced key immunological markers including plasma PO, LZM, and hemocyanin activities, and hepatopancreas *hemo* expression. These results align with previous researches in rainbow trout [29], Nile tilapia [64], beluga sturgeon [75], and white shrimp [21,76], consistently indicating the superiority of organic trace minerals. A key aspect of the underlying mechanism may be that organic Zn is more easily absorbed by cells, and Zn acts as a second messenger for various cellular activities, promoting immune homeostasis and functional signaling pathway transduction through the Zn-Zn transport axis [77].

The intestinal microbiota of aquatic animals is closely linked to host health, with its composition being influenced by feed, environment, and host physiology [78,79]. This study identified Proteobacteria, Firmicutes, Bacteroidetes, and Actinobacteria as the four dominant phyla in shrimp intestines, consistent with previous studies [27,47]. Although alpha diversity showed no significant differences, PCoA revealed distinct clustering of organic minerals treatments from control and IM100 groups and more differential bacteria were observed in the organic trace mineral group, indicating a regulatory effect on the shrimp intestinal microbial community. Recent studies suggest organic minerals may shape healthier microbial communities. Organic Mn might facilitate the formation of a healthier intestinal microbiota community by regulating the competition for Mn^2+^ between the host and pathogens [26]. Study in Nile tilapia shown that metal-amino acid complexes (Zn, Se, Cu, Fe, and Mn) effectively increase probiotics abundance and suppress pathogenic bacteria [80]. Specifically, diet OM50 significantly increased the relative abundance of probiotics *Pseudomonas* and *Enterococcus*, and significantly decreased the relative abundance of potential pathogenic bacteria *Vibrio* and *Planctomicrobium*. Gram et al. reported that the mortality rate of rainbow trout inoculated with *Pseudomonas* was 22% lower than that of non-inoculated fish [81]. Furthermore, adding *Enterococcus* to the feed can reduce the relative abundance of potential pathogenic bacteria and enhance the health of shrimp [82]. Diet OM50 resulted in lower relative abundances of potential pathogenic bacteria such as *Vibrio* and *Planctomicrobium*. *Vibrio* is one of the most severe bacterial diseases in shrimp farming and is the fundamental cause of the early mortality syndrome [83]. *Planctomicrobium* is related to host diseases and has been shown to exhibit resistance to various antibiotics [84]. At present, the specific mechanism through which the addition of organic trace minerals contributes to these microbiome changes remains unclear. However, we propose the following two possible mechanisms: Firstly, organic trace minerals may enhance the nutritional status and immune function of the host, thereby creating a more favorable environment for the growth and colonization of probiotics while suppressing the survival and reproduction of pathogenic bacteria. Secondly, trace minerals may directly impact the metabolic pathways and ecological balance of the microbial community, facilitating metabolic processes beneficial to the growth of probiotics and inhibiting those detrimental to the growth of pathogenic bacteria. The precise mechanism still requires further exploration and verification.

## 5. Conclusions

In this study, shrimp consuming organic trace minerals are able to absorb and utilize these critical nutrients with greater efficiency. In addition to enhanced mineral accumulation, shrimp fed organic trace minerals exhibited marked improvements in lipid metabolism, antioxidant capacity, and immune response. The study further revealed the beneficial effects of organic trace minerals on the intestinal microbiota of shrimp, which may play a crucial role in promoting overall shrimp health. Consequently, for shrimp, the use of organic trace minerals at lower inclusion rates is a beneficial choice in diet formulation.

## Figures and Tables

**Table 1:** Formulation and proximate composition of experimental diets.

Ingredients (%)	Groups				
	Control	IM100	IM50	OM50	OM33
Flour	24.19	24.19	24.19	24.19	24.19
Soybean meal	24.00	24.00	24.00	24.00	24.00
Peanut meal	15.00	15.00	15.00	15.00	15.00
Dephenolized cottonseed protein	14.00	14.00	14.00	14.00	14.00
Fish meal	7.00	7.00	7.00	7.00	7.00
Shrimp meal	1.00	1.00	1.00	1.00	1.00
gluten	4.00	4.00	4.00	4.00	4.00
Chicken meal	3.00	3.00	3.00	3.00	3.00
Fish oil	4.00	4.00	4.00	4.00	4.00
Phospholipid	2.00	2.00	2.00	2.00	2.00
Crystal methionine	0.30	0.30	0.30	0.30	0.30
Monocalcium phosphate	1.00	1.00	1.00	1.00	1.00
Choline chloride ^1^	0.20	0.20	0.20	0.20	0.20
Y_2_O_3_^1^	0.01	0.01	0.01	0.01	0.01
Vitamin mix ^2^	0.10	0.10	0.10	0.10	0.10
Mineral mix ^3^	0.20	0.20	0.20	0.20	0.20
Analyzed Nutrient Compositions (% Dry Matter)					
Moisture	5.21	5.92	6.79	6.49	5.28
Crude protein	47.13	47.11	46.82	46.98	46.19
Crude lipid	4.93	5.06	4.27	4.77	4.58
Ash	8.00	7.07	6.15	6.78	6.74
Analyzed Trace Minerals Contents (mg/kg; Dry Matter)					
Zn	45.04	155.17	95.45	98.38	79.37
Cu	5.56	29.05	15.03	17.08	13.15
Mn	37.36	55.12	43.73	51.26	39.23
Fe	209.57	236.08	176.13	181.17	171.78

^1^ Choline chloride and Y_2_O_3_ were purchased from Shanghai Macklin Biochemical Co., Ltd., Shanghai, China. ^2^ Vitamin premix was purchased from DSM Vitamins (Sichuan) Ltd., Chengdu, China. ^3^ Mineral contained (/kg diet, dry matter): Mg, 125 mg; I, 0.5 mg; Co, 0.5 mg.

**Table 2:** Growth performance of shrimp fed with organic or inorganic trace minerals premixes.

Diets	Control	IM100	IM50	OM50	OM33
IBW (g)	7.19 ± 0.08	7.21 ± 0.01	7.22 ± 0.07	7.27 ± 0.07	7.17 ± 0.05
FBW (g)	14.75 ± 0.26	15.45 ± 0.21	14.91 ± 0.24	15.41 ± 0.26	14.41 ± 0.26
WGR (%)	107.83 ± 2.58	114.46 ± 3.12	106.77 ± 4.12	112.21 ± 5.39	101.18 ± 4.38
SGR (%/day)	1.31 ± 0.0221	1.36 ± 0.0259	1.30 ± 0.0358	1.34 ± 0.0452	1.25 ± 0.0385
CF (100 g/cm^3^)	1.94 ± 0.0052	1.92 ± 0.0255	1.92 ± 0.0049	1.94 ± 0.0114	1.91 ± 0.0089
FI (%/day)	2.44 ± 0.0317	2.41 ± 0.0234	2.40 ± 0.0284	2.41 ± 0.0212	2.44 ± 0.0263
FE	0.49 ± 0.0304	0.54 ± 0.0145	0.52 ± 0.0175	0.53 ± 0.0207	0.49 ± 0.0175
Survival (%)	86.25 ± 5.45	81.88 ± 1.57	85.00 ± 2.04	81.25 ± 1.61	86.25 ± 2.60

Results are shown as means ± S.E. of four replicate tanks.

**Table 3:** Plasma biochemical constituents of shrimp fed with organic or inorganic trace minerals premixes.

Diets	Control	IM100	IM50	OM50	OM33
T-CHO (mmol/L)	17.26 ± 0.58 ^a^	9.05 ± 0.41 ^c^	14.75 ± 1.13 ^ab^	12.27 ± 0.46 ^b^	13.94 ± 0.88 ^b^
TG (mmol/L)	3.53 ± 0.10 ^a^	1.19 ± 0.07 ^c^	2.40 ± 0.10 ^b^	1.36 ± 0.11 ^c^	1.53 ± 0.10 ^c^
GLU (mg/dL)	31.86 ± 1.83	32.90 ± 1.93	31.81 ± 1.11	32.24 ± 1.15	32.67 ± 0.70
TP (g/L)	39.32 ± 1.38 ^c^	59.13 ± 1.46 ^a^	49.31 ± 1.77 ^b^	64.31 ± 2.69 ^a^	44.20 ± 1.76 ^bc^
ALB (g/L)	23.06 ± 0.88 ^ab^	27.80 ± 1.96 ^a^	25.82 ± 2.51 ^ab^	27.84 ± 2.05 ^a^	18.22 ± 1.75 ^b^

Results are shown as means ± S.E. of four replicate. Different superscript letters in each row indicate significant differences (*p* < 0.05). T-CHO, total cholesterol; TG, triglyceride; GLU, glucose; TP, total protein; ALB, albumin.

**Table 4:** Effects of organic or inorganic trace mineral premixes on ADC of trace minerals in white shrimp.

Diets	Control	IM100	IM50	OM50	OM33
Zn	25.91 ± 2.33 ^a^	11.68 ± 1.17 ^b^	20.98 ± 1.33 ^a^	22.36 ± 2.50 ^a^	18.92 ± 0.51 ^ab^
Cu	82.29 ± 1.70 ^b^	85.90 ± 0.77 ^ab^	89.59 ± 0.94 ^a^	89.40 ± 0.90 ^a^	87.79 ± 0.50 ^a^
Mn	−748 ± 173	−422 ± 168	−869 ± 195	−390 ± 103	−506 ± 178
Fe	−165 ± 75	−73 ± 16	−306 ± 120	−178 ± 73	−75 ± 7

Results are shown as means ± S.E. of four replicate. Different superscript letters in each row indicate significant differences (*p* < 0.05).

## Data Availability

The data of 16S rRNA sequence presented in the study are deposited in https://www.ncbi.nlm.nih.gov/sra (accessed on 12 March 2025), under Accession Number PRJNA1235146.

## Supplementary Materials

The following supporting information can be downloaded at: https://www.mdpi.com/article/10.3390/biology14050540/s1, Figure S1: The rarefaction curves and the species accumulation boxplot of intestinal microbiota; Figure S2: Mata-statistical analysis of the intestinal microbiota of white shrimp at the genus level in the control group and the IM100 (A), IM50 (B), OM50 (C), and OM33 (D) groups respectively. Table S1: Primers and amplification information of qPCR; Table S2: Effects of organic or inorganic trace mineral premixes on Zn, Cu, Mn, and Fe accumulation (mg/kg, dry matter) in various tissues; Table S3: Effects of organic or inorganic trace minerals premixes on metal transporter genes in the hepatopancreas of white shrimp; Table S4: Effects of organic or inorganic trace minerals premixes on antioxidant capacity of white shrimp; Table S5: Effects of organic or inorganic trace minerals premixes on immune response of white shrimp; Table S6: Alpha diversity index of microbial community of intestine.
