# Supplementary material for: Organic Trace Mineral Source Enhances the Bioavailability, Health Status, and Gut Microbiota Community in White Shrimp (Penaeus vannamei)
Source: Biology (Basel). 2025 May 13;14(5):540. doi: 10.3390/biology14050540 (PMC12108777; doi:10.3390/biology14050540)
Supplement: Supplementary file 1 [file biology-14-00540-s001.zip › biology-3554422-supplementary.pdf]

Organic Trace Mineral Source Enhances the Bioavailability, Health Status, and Gut Microbiota Community in White Shrimp (*Penaeus vannamei*)

Weijian Huang [1], Jinzhu Yang [1], Xiao Li [1], Gang Lin [2], Mingzhu Li [3], Yanjiao Zhang [1,4,]* and Kangsen Mai [1,4]

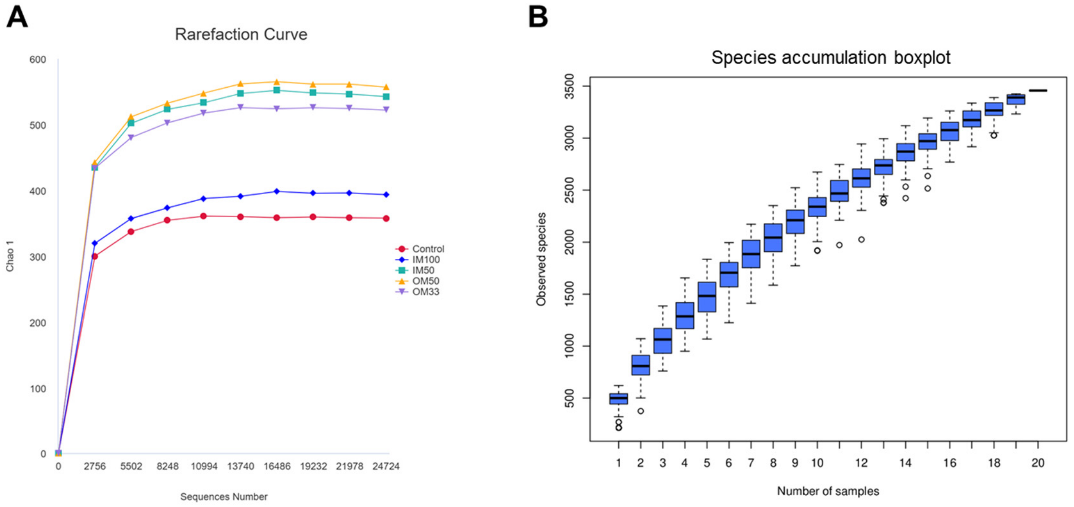

Figure S1. The rarefaction curves (A) and the species accumulation boxplot (B) of intestinal microbiota.

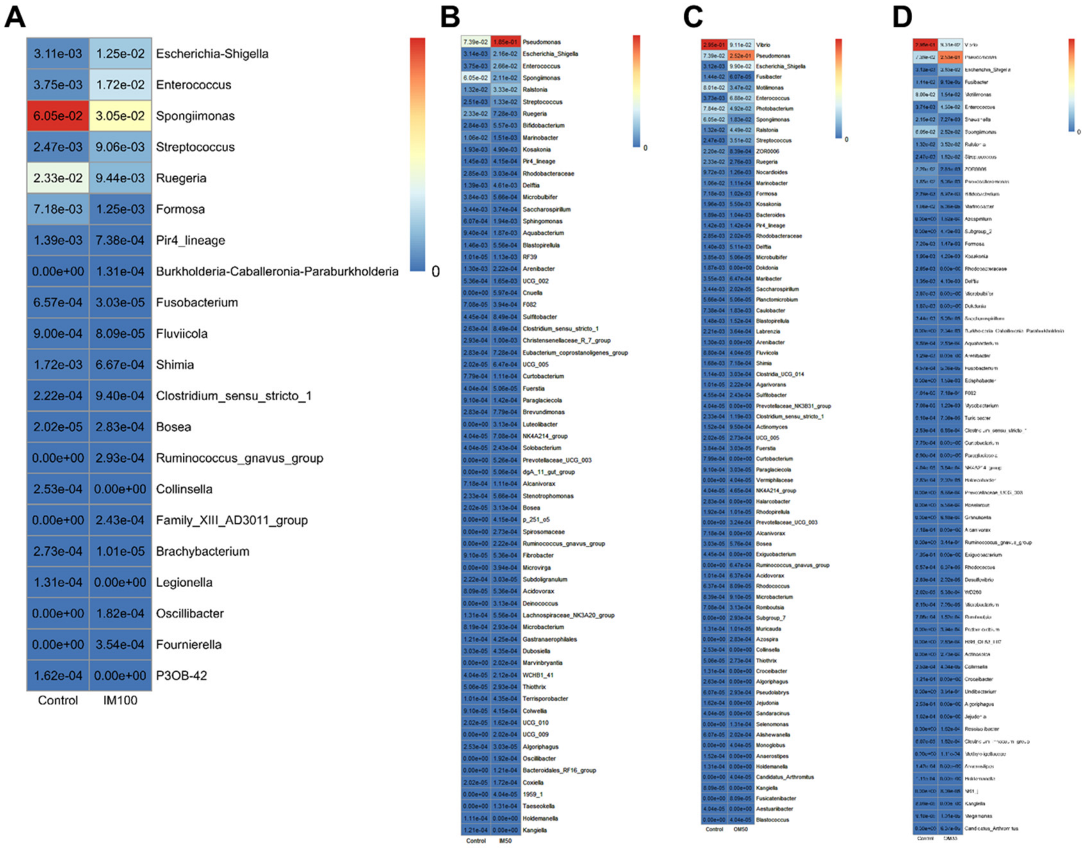

Figure S2. Mata-statistical analysis of the intestinal microbiota of white shrimp at the genus level in the control group and the IM100 (A), IM50 (B), OM50 (C), and OM33 (D) groups respectively. Results are shown as means ± S.E. of 4 replicate. The values in the cells represent the relative abundance of the microbiota. The relative abundances of the microbiota shown in the figure all exhibited statistically significant differences ($P < 0.05$).

Table S1. Primers and amplification information of qPCR.

Genes	Primer sequence (5'-3')	Product length	Amplification efficiency	GenBank Accession no.
ZIP1	F: TGTCGGGTGCCTGTGCTT<br>R: GGTGGTGCTGGTTGTGGTG	227	1.004	XM_027360656.1
ZIP11	F: ATCTCCAGTAACCCAAGC<br>R: GTGCCAGAAGTGACCAA	170	1.059	XM_027361506.1
ZIP14	F: TTGGTCATAAAGAGCGTCCCA<br>R: GCTACTGCTTGCCTCATGGT	277	1.054	XM_027375334.1
ZnT2	F: TCTTCAGCGACCTTACATC<br>R: ACACTCACAACTGCACCC	124	1.014	XM_027377440.1
ZnT6	F: GCAAGAATACCTGGAACA<br>R: AGAGTCACAGATGCGAAC	368	0.985	XM_027372522.1
ZnT7	F: CCAGTAAGCAGGACGAA<br>R: AGCCAGGATGAAGAAGG	339	0.971	XM_027353047.1
Ctr1	F: AAGCAACAGCCATACGC<br>R: GCAGGAAGAATCGCCAC	118	0.993	XM_027362143.1
ATOX1	F: TCAGGTTGTTAGACGGCAGC<br>R: AGGAGAGAGTGCTGGTCACA	187	0.949	XM_027376680.1
ATP7b	F: GATAAAGGGAGCAGAACC<br>R: CTGGCAGAACTTGGAGAT	217	1.015	XM_027356375.1
MT	F: AGAAATGCGTCTGTGCCG<br>R: CATGGGTTGGTGCATCTTG	139	1.004	JN707684.1
acp	F: CCTGGAAGAATGGGACT<br>R: CATCTGTTCGTGGTTGC	254	1.016	KR676449.1
akp	F: TAGAAGGACTGGGCTACTGG<br>R: GGCGGTCAGAGTGGAGAT	137	1.006	KR534873.1
proPO	F: ACCAGCAGCGTCTTCTTTAC<br>R: GGAGGCGTATCATCGTTCT	106	0.959	AY723296.1
cat	F: TAAGGGAGCAGGTGCCTTTG<br>R: ATCCCTGGCAGTGTCAGTTG	157	0.938	AY518322.1
gpx	F: GGCACCAGGAGAACACTACC<br>R: TCGAAGTTGTTCCCAGGACG	73	1.017	AY973252.2
hemo	F: AGACTGGGCATCCTTTGTCG<br>R: TCATAGAGGGGAGGGAGCAC	135	0.940	MK896907.1
β-actin	F: CGAGAGGAAGCAGCACGTA<br>R: GACGATGGAGGGGAACACAG	164	0.974	AF300705.2

Abbreviations: *zip1*, zinc transporter ZIP1-like; *zip11*, zinc transporter ZIP11-like; *zip14*, zinc transporter ZIP14-like; *znt2*, zinc transporter 2-like; *znt6*, zinc transporter 6-like; *znt7*, zinc transporter 7-like; *ctr1*, high affinity copper uptake protein 1-like; *ATOX1*, copper transport protein ATOX1-like; *ATP7b*, copper-transporting ATPase 2-like; *MT*, metallothionein; *acp*, acid phosphatase; *akp*, alkaline phosphatase; *proPO*,

pro-phenoloxidas; *Hemo*, hemocyanin; *cat*, catalase; *gpx*, glutathione peroxidase.

Table S2. Effects of organic or inorganic trace mineral premixes on Zn, Cu, Mn, and Fe accumulation (mg/kg, dry matter) in various tissues.

Diets	Control	IM100	IM50	OM50	OM33
**Zn**					
Whole body	44.11 ± 0.35^{c}	54.68 ± 0.09^{ab}	51.87 ± 0.65^{b}	56.34 ± 0.28^{a}	54.21 ± 0.13^{b}
Muscle	28.68 ± 0.15^{c}	32.18 ± 0.29^{a}	32.16 ± 0.55^{abc}	31.57 ± 0.79^{abc}	30.25 ± 0.21^{b}
Hepatopancreas	44.78 ± 3.68^{d}	95.57 ± 2.23^{b}	80.22 ± 0.54^{c}	111.20 ± 3.87^{a}	108.04 ± 2.07^{a}
Plasma	3.30 ± 0.42^{c}	8.43 ± 0.67^{a}	5.15 ± 0.45^{bc}	5.42 ± 0.15^{b}	5.03 ± 0.25^{bc}
Shell	12.69 ± 0.83^{c}	16.57 ± 0.66^{b}	21.35 ± 0.30^{a}	21.57 ± 0.62^{a}	17.53 ± 0.44^{b}
Feces	108 ± 12^{d}	371 ± 9^{a}	225 ± 8^{bc}	233 ± 16^{b}	184 ± 7^{c}
**Cu**					
Whole body	43.21 ± 0.84^{c}	68.62 ± 0.89^{a}	55.36 ± 2.41^{b}	63.07 ± 3.58^{ab}	54.91 ± 1.97^{b}
Muscle	18.85 ± 0.36^{d}	30.45 ± 0.22^{a}	27.04 ± 0.68^{b}	26.69 ± 0.29^{b}	22.48 ± 0.49^{c}
Hepatopancreas	18.80 ± 0.87^{c}	82.84 ± 4.50^{a}	27.23 ± 1.98^{bc}	35.96 ± 1.62^{b}	24.21 ± 1.04^{c}
Plasma	37.32 ± 1.36^{c}	55.68 ± 1.43^{a}	41.17 ± 1.59^{bc}	46.29 ± 1.94^{b}	37.70 ± 1.49^{c}
Shell	22.77 ± 1.57^{b}	32.96 ± 0.51^{a}	39.71 ± 2.60^{a}	38.32 ± 3.33^{ab}	30.09 ± 0.67^{ab}
Feces	2 ± 1^{c}	11 ± 1^{a}	4 ± 0^{bc}	5 ± 1b	$5 \pm 0b^{c}$
**Mn**					
Whole body	31.15 ± 1.03	29.95 ± 2.94	31.09 ± 4.74	34.33 ± 4.52	27.62 ± 3.26
Muscle	14.60 ± 0.72^{a}	10.16 ± 0.71^{ab}	11.13 ± 1.59^{ab}	9.94 ± 1.30^{b}	9.29 ± 0.13^{b}
Hepatopancreas	36.51 ± 3.10^{a}	10.97 ± 0.73^{b}	18.77 ± 2.33^{b}	17.09 ± 1.66^{b}	15.17 ± 1.31^{b}
Plasma	4.04 ± 0.01^{b}	5.95 ± 0.02^{a}	4.03 ± 0.01^{b}	4.01 ± 0.01^{b}	4.01 ± 0.01^{b}
Shell	38.90 ± 3.69^{b}	47.00 ± 3.60^{ab}	42.83 ± 3.11^{ab}	56.13 ± 1.70^{a}	36.24 ± 4.07^{b}
Feces	887 ± 183	768 ± 243	1173 ± 192	707 ± 126	661 ± 188
**Fe**					
Whole body	40.10 ± 2.37^{abc}	34.06 ± 4.09^{bc}	44.35 ± 1.36^{ab}	47.78 ± 1.87^{a}	30.96 ± 2.01^{c}
Muscle	37.96 ± 2.58^{ab}	42.79 ± 0.79^{ab}	31.78 ± 2.70^{b}	47.28 ± 0.34^{a}	36.72 ± 4.23^{ab}
Hepatopancreas	251.65 ± 12.11^{a}	216.43 ± 4.28^{a}	174.07 ± 6.09^{b}	142.38 ± 8.32^{bc}	122.08 ± 1.31^{c}
Plasma	18.58 ± 0.91^{b}	30.59 ± 2.16^{a}	18.89 ± 0.57^{b}	19.71 ± 0.80^{b}	17.95 ± 0.66^{b}
Shell	70.67 ± 6.29	54.91 ± 3.93	56.12 ± 5.67	58.73 ± 7.64	55.25 ± 2.76
Feces	1601 ± 517	1091 ± 87	1961 ± 512	1418 ± 340	848 ± 33

Results are shown as means ± S.E. of 4 replicate. Different superscript letters in each row indicate significant differences ($P < 0.05$).

Table S3. Effects of organic or inorganic trace minerals premixes on metal transporter genes in the hepatopancreas of white shrimp.

Diets	Control	IM100	IM50	OM50	OM33
ZIP1	1.00 ± 0.07	1.07 ± 0.10	1.23 ± 0.07	1.12 ± 0.17	1.10 ± 0.17
ZIP11	1.14 ± 0.13	1.55 ± 0.05	1.21 ± 0.16	1.39 ± 0.09	1.33 ± 0.17
ZIP14	1.00 ± 0.03^{c}	1.31 ± 0.08^{b}	1.31 ± 0.08^{b}	1.62 ± 0.05^{a}	1.47 ± 0.07^{ab}
ZnT2	1.00 ± 0.02^{b}	1.94 ± 0.08^{a}	1.80 ± 0.09^{a}	2.14 ± 0.07^{a}	2.08 ± 0.10^{a}
ZnT6	0.99 ± 0.03^{b}	1.22 ± 0.13^{ab}	1.01 ± 0.03^{b}	1.44 ± 0.01^{a}	1.16 ± 0.05^{ab}
ZnT7	1.05 ± 0.02	1.03 ± 0.01	1.00 ± 0.03	1.08 ± 0.03	1.05 ± 0.01
Ctr1	1.01 ± 0.10^{b}	2.57 ± 0.43^{a}	2.05 ± 0.08^{ab}	2.63 ± 0.32^{a}	2.84 ± 0.17^{a}
ATOX1	1.02 ± 0.11	1.18 ± 0.08	1.23 ± 0.08	1.25 ± 0.19	1.26 ± 0.06
ATP7b	1.01 ± 0.08^{b}	2.31 ± 0.15^{a}	2.18 ± 0.10^{a}	2.15 ± 0.12^{a}	2.46 ± 0.10^{a}
MT	1.00 ± 0.05^{c}	1.51 ± 0.05^{b}	1.46 ± 0.09^{b}	2.07 ± 0.07^{a}	1.52 ± 0.07^{b}

Results are shown as means ± S.E. of 4 replicate. Different superscript letters in each row indicate significant differences ($P < 0.05$).

Table S4. Effects of organic or inorganic trace minerals premixes on antioxidant capacity of white shrimp.

Diets	Control	IM100	IM50	OM50	OM33
**Enzyme**					
T-SOD	20.61 ± 0.64[c]	56.06 ± 1.95[a]	26.21 ± 2.80[bc]	53.13 ± 2.54[a]	30.65 ± 1.64[b]
GPX	499 ± 13[c]	691 ± 15[ab]	625 ± 15[b]	726 ± 9[a]	718 ± 22[a]
MDA	275.00 ± 7.22[a]	103.13 ± 3.13[c]	159.38 ± 5.98[b]	106.25 ± 6.25[c]	150.00 ± 7.22[b]
**Genes**					
cat	1.03±0.10[b]	2.07±0.26[a]	1.55±0.13[ab]	2.08±0.27[a]	2.18±0.15[a]
gpx	1.01±0.08[b]	1.71±0.10[a]	1.49±0.17[ab]	1.67±0.02[a]	1.65±0.14[a]

Results are shown as means ± S.E. of 4 replicate. Different superscript letters in each row indicate significant differences ($P < 0.05$).

Table S5. Effects of organic or inorganic trace minerals premixes on immune response of white shrimp.

Diets	Control	IM100	IM50	OM50	OM33
**Enzyme**					
ACP	20.33 ± 0.32^{b}	25.16 ± 0.73^{a}	19.09 ± 0.71^{b}	19.51 ± 0.89^{b}	20.98 ± 0.36^{b}
AKP	23.88 ± 0.89^{c}	33.71 ± 2.30^{ab}	33.27 ± 2.30^{ab}	35.10 ± 1.82^{a}	27.00 ± 0.79^{bc}
PO	21.65 ± 0.59^{b}	30.07 ± 2.33^{a}	23.72 ± 1.06^{b}	29.24 ± 0.61^{a}	25.16 ± 0.48^{ab}
LZM	31.11 ± 2.38^{ab}	39.89 ± 2.87^{ab}	33.56 ± 0.25^{b}	44.28 ± 0.74^{a}	40.39 ± 1.87^{ab}
Hemocyanin	284 ± 1^{c}	333 ± 0^{a}	288 ± 3^{c}	347 ± 5^{ab}	321 ± 1^{b}
**Genes**					
acp	1.01 ± 0.06^{b}	1.78 ± 0.10^{a}	2.00 ± 0.11^{a}	1.92 ± 0.00^{a}	1.30 ± 0.04^{b}
akp	1.00±0.05	1.21±0.07	1.20±0.10	1.37±0.11	1.23±0.15
proPO	1.01±0.08	1.14±0.18	1.30±0.16	1.30±0.18	0.94±0.06
Hemo	1.01 ± 0.06^{b}	3.98 ± 0.31^{a}	2.93 ± 0.09^{a}	4.26 ± 0.66^{a}	3.15 ± 0.45^{a}

Results are shown as means ± S.E. of 4 replicate. Different superscript letters in each row indicate significant differences ($P < 0.05$).

Table S6. Alpha diversity index of microbial community of intestine.

Diets	Control	IM100	IM50	OM50	OM33
ASVs	356 ± 63^{b}	383 ± 40^{ab}	530 ± 31^{a}	543 ± 28^{a}	513 ± 20^{ab}
Chao1	358 ± 64^{b}	393 ± 42^{ab}	542 ± 32^{a}	556 ± 27^{a}	522 ± 23^{ab}
Goods_coverage	0.9995 ± 0.0002^{a}	0.9987 ± 0.0004^{ab}	0.9980 ± 0.0002^{b}	0.9977 ± 0.0002^{b}	0.9986 ± 0.0004^{ab}
Pielou_e	0.6238 ± 0.0083	0.5764 ± 0.0215	0.5602 ± 0.0119	0.5492 ± 0.0199	0.6113 ± 0.0448
Shannon	5.24 ± 0.16	4.93 ± 0.21	5.07 ± 0.12	4.99 ± 0.22	5.51 ± 0.43
Simpson	0.9312 ± 0.0060	0.9058 ± 0.0100	0.9136 ± 0.0061	0.8982 ± 0.0115	0.9109 ± 0.0254

Results are shown as means ± S.E. of 4 replicate. Different superscript letters in each row indicate significant differences ($P < 0.05$).
